# Comparative evaluation of intraoral scanners and a spectrophotometer for percent correct shade identification in clinical dentistry

**DOI:** 10.1007/s00784-024-06124-0

**Published:** 2025-01-02

**Authors:** Sascha Hein, Julian Nold, Matthias Masannek, Stephen Westland, Benedikt C. Spies, Karl Thomas Wrbas

**Affiliations:** 1 Graduate School of Colour Science and Technology, School of Design, University of Leeds, Woodhouse Lane, Leeds, LS2 9JT UK; 2 Center for Dental Medicine, Department of Operative Dentistry and Periodontology, Faculty of Medicine and Medical Center, University of Freiburg, Hugstetter Str. 55, 79106 Freiburg im Breisgau, Germany; 3 Center for Dental Medicine, Department of Prosthetic Dentistry, Faculty of Medicine, University of Freiburg, Hugstetter Str. 55, 79106 Freiburg im Breisgau, Germany; 4 Faculty of Medicine and Dentistry, Danube Private University, Steiner Landstraße 124, Krems an der Donau, 3500 Austria

**Keywords:** Intraoral scanners, Shade matching, Color measurement, Shade guide reliability, Visual-instrumental agreement

## Abstract

**Objectives:**

The study aimed to assess the percent correct shade identification of four intraoral scanners (IOS) and a spectrophotometer, focusing on how reliably each device selects the correct tooth shade compared to a visual observer’s selection. The research question addresses how much clinicians can trust the device-selected shade without visual verification.

**Materials and methods:**

Sixteen participants with natural, unrestored teeth were included. The teeth evaluated were tooth 21 (left maxillary central incisor), tooth 23 (left maxillary canine), and tooth 26 (first left maxillary molar). Tooth color was measured using four IOS devices and the Vita Easyshade V in three regions: incisal, middle, and cervical. The nearest 3D Master shade selected by each device was compared to the visual observer’s selection. The percent exact match, acceptable match (> 1.2, ≤ 2.7 ∆*E*_ab_), and mismatch type A (< 2.7, ≤ 5.4 ∆*E*_ab_) were calculated. Statistical analysis was performed using a chi-square test with a 95% confidence level.

**Results:**

The overall clinical pass rate was highest for Carestream (78.2%), followed by Easyshade (63.5%), Primescan (51.2%), Trios (39.5%), and Medit (31.3%). Carestream also recorded the highest rate of mismatch type A (47.7%). Significant differences between devices were observed for all categories (*p* < 0.05).

**Conclusions:**

Carestream demonstrated the highest overall clinical pass rate, while Medit exhibited the lowest. The study highlights the variability between devices in shade matching performance.

**Clinical relevance:**

This study highlights the importance of considering device performance when relying on IOS or spectrophotometers for shade selection without visual assessment, as the reliability can vary significantly across devices.

## Introduction

Achieving accurate shade matching in dentistry poses a significant challenge for restorative teams [1], with color mismatches frequently leading to esthetic issues [2] and substantial costs [3]. Visual shade selection remains the most widely used method in dentistry; however, it is often subjective and inconsistent [4]. Various observer-related factors, including gender [5–7], experience [8, 9] and color vision deficiencies [10], can significantly affect its reliability. Among environmental factors, the type and quality of lighting in the dental setting play a critical role in the accuracy of visual shade matching [11]. Recent research has highlighted the extensive variability in natural tooth color, identifying 1,173 unique, visually distinguishable shades—a diversity that current shade guides fail to fully encompass [12]. Additional factors such as geographic location, gender, age, and ethnicity have also been shown to influence natural tooth color [13].

Due to these complexities, instrumental shade measurement has garnered increasing interest, prompting a growing focus on evaluating the accuracy and precision of shade measurement devices [14, 15], with recent attention given to intraoral scanners (IOS) [16–19]. These devices are becoming more essential in restorative dentistry, with claims that they can also accurately measure tooth shades [20, 21].

In clinical dentistry, the terms *accuracy* and *precision* are often used interchangeably, though their meanings can differ from how they are understood in color science. For example, an impression is said to be *accurate*, while there may be discussions of *marginal precision* in relation to indirect restorations or tooth anatomy replication [22]. In these contexts, *accuracy* and *precision* often imply a high level of congruency between the desired outcome and what was achieved, leading to their frequent interchangeable use.

However, in color science, these terms are strictly defined, which can lead to confusion in dental colorimetric research. Colorimetric uncertainty is separated between accuracy and precision. Colorimetric accuracy refers to the calculated color difference between the spectral reflectance factors of reference standards, such as a set of 12 ceramic tiles, and the corresponding measurements from a given test device [23–25]. Colorimetric precision on the other hand refers to how consistently a measuring device provides results [26]. It is assessed by calculating the average color differences between 30 recommended repeated measurements of the same reference standards under identical conditions while colorimetric reproducibility measures consistency when certain conditions, such as the operator or sample, are varied [27, 28].

Performing proper assessments of accuracy and precision in the context of instrument profiling is not a trivial task [29, 30] and may not even be feasible for many shade measurement devices used in dentistry. These devices often rely on a mix of measurement technologies combined with illumination geometries outside of those recommended by the *Commission internationale de l’éclairage* (CIE), to facilitate easy operation and meet clinical requirements.

Numerous studies have set out to investigate *supposed* device accuracy [31], often by designating a spectrophotometer, most commonly the Vita Easyshade, as the *gold standard* [32–35], assuming it measures the *true* colorimetric values. The computed color difference between a set of tooth-colored samples measured by a test device and the reference device is frequently misinterpreted as colorimetric accuracy when it would be more accurately described as *inter-device agreement* [36]. Other studies have aimed to count how often a test device’s selected shade matched the visual shade selection by an experienced observer, reporting the results as *accuracy* [4, 37]. However, this approach would be more appropriately termed *percent matching shade identification*.

Nevertheless, evaluating the congruency between observer- and instrument-selected shades offers practical insights into how much clinicians can rely on established shade measurement devices and, increasingly, on IOS, especially when visual shade selection was not performed due to the demands of clinical practice.

Therefore, the aim of this study was to evaluate the reliability of shade selection by four contemporary IOS and one spectrophotometer, comparing device performance against an expert visual observer. The primary objective was to assess the clinical reliability of each device in shade selection by gathering data on the clinical pass rate, indicating how much clinicians can depend on the device’s shade selection in practice. The null hypothesis was that there is no difference in device performance.

## Materials and methods

### Study setting

For this study, a proportional ethical review application was submitted and received approval from the local Ethics Committee of the Medical Faculty (Approval number: EK-Freiburg 21-1169). The study followed good clinical practice guidelines and adhered to the principles outlined in the Declaration of Helsinki. A total of 16 participants, male and female, all under the age of 35 and with natural, unrestored teeth, were included. Participants were instructed to maintain high dental hygiene prior to their appointment, which was verified by the dentist to ensure all measurements were performed on clean teeth. The teeth evaluated in this study were tooth 21 (left maxillary central incisor), tooth 23 (left maxillary canine), and tooth 26 (first left maxillary molar). Participant data was collected anonymously to protect their privacy, and only the project management team had access to the full data sets, ensuring no direct patient identification.

### Study procedure

The devices examined in this study, listed in Table 1, include four contemporary IOS and the Vita Easyshade V. All devices were operated in accordance with the manufacturers’ recommendations, following specified scanning procedures and calibration protocols.

**Table 1:** Devices included in the study, consisting of four IOS and Vita Easyshade V, along with corresponding abbreviations for each device

Device Name	Manufacturer	Location	Abbreviation	Software
Primescan	Dentsply Sirona	Bensheim, Germany	‘Primescan’	Cerec SW 5.2.10
Medit i700	Medit	Seoul, South Korea	‘Medit’	Medit Link 3.1.4
CS3700	Carestream LLC	Atlanta, USA	‘Carestream’	Dexis 1.0.10.902
Trios 3	3Shape	Copenhagen, Denmark	‘Trios’	Trios A/S 22.1.3
Easyshade V	Vita Zahnfabrik	Bad Säckingen, Germany	‘Easyshade’	ES_Helper 1.0.11081.369

A single expert observer, with seven years of experience as a dental technician and three years of experience as a dentist, conducted the visual shade assessments. The observer utilized a 3D Master shade guide (LOT J017B027IO, VITA Zahnfabrik, Bad Säckingen, Germany) for visual shade selections in three regions: the incisal, middle, and cervical areas of the labial and buccal surfaces. During each assessment, the patient sat upright, facing the observer. The lighting environment was optimized for shade selection, featuring large north-facing windows providing natural light, supplemented by color-corrected ceiling lighting with an average color temperature of 5000 K to 6500 K and an illuminance of 1000 to 1500 lx, depending on the time of day (08:30 to 17:00, summer time). The walls were painted in a neutral light grey to minimize color interference.

### Color measurement

Color measurements were performed by the same, trained operator over several days. Each scan captured all teeth in the upper jaw, while the lower jaw was not scanned. Easyshade was used to measure tooth color in the incisal, medial, and cervical areas of the labial and buccal surfaces. Each IOS employed the color measurement mode of its respective system software to obtain shade designations in approximately the same three regions. In both cases, 3D Master shade designations were selected and recorded. Each complete measurement sequence took less than two minutes. To minimize potential color changes from dehydration, patients were asked to rinse their mouths with room temperature water to rehydrate their teeth between measurements.

### Computation of tooth color

The spectral data from Easyshade, covering 400 to 700 nm in 10 nm intervals, was processed using the manufacturer’s *ES-Helper* software and converted to the CIELAB color space which is a standardized system developed by the *Commission Internationale de l’Éclairage* (CIE) for describing and quantifying color. It represents color in three coordinates: L* for lightness, which ranges from 0 (black) to 100 (white); a* for the green-red axis, with negative values indicating green and positive values indicating red; and b* for the blue-yellow axis, with negative values indicating blue and positive values indicating yellow. CIELAB coordinates were computed under Illuminant D65 and the CIE 1931 standard colorimetric observer [38]. The same process was applied to the 3D Master shade guide used for visual assessments to ensure consistent data. The choice of file format (OBJ for Primescan and Medit, PLY for Carestream and Trios) was determined by the standard export capabilities of the respective devices. These formats were not selected by preference but reflect the default outputs provided by the devices. Both OBJ and PLY formats are widely used in 3D rendering and they are fully compatible with MeshLab (version 2023.12), the software used to process and visualize the intraoral scans in this study. Scans were 3D-rotated to capture labial/buccal views, ensuring that measurements were consistently taken in the same three regions for both the natural teeth and all shade tabs of the Vita 3D Master shade guide. A custom MATLAB (R2023b; MathWorks, Natick, MA, USA) routine was used to capture average sRGB values from tooth surfaces in each scan, which were converted to XYZ and CIELAB coordinates using MATLAB’s color toolbox. The resulting data included the average tooth color from Easyshade and each IOS, as well as the corresponding CIELAB values for the nearest shade guide match.

### Percent correct shade identification

Using the CIELAB values of the natural target tooth for each region and the nearest device-selected shade tab for the same regions, the extent of the discrepancy between the device’s selection and the visually selected shade was calculated in cases where the two differed. This resulted in a total of 315 CIELAB values for comparisons per device.

In this study, the ∆*E*_ab_ formula was chosen over the more complex ∆*E*_00_ because a recent multicenter study showed that the basic Euclidean distance provided better visual-instrumental agreement in the CIELAB region relevant to natural tooth colors [39].

Percent correct shade identification was determined in three categories: Exact Match, Acceptable Match, and Mismatch Type A. Exact Match refers to instances where the device selected the same shade as the visual observer, while Acceptable Match indicates a clinically acceptable color difference (> 1.2, ≤ 2.7 Δ*E*_ab_), between the device-selected shade and the target tooth. Mismatch Type A represents cases where the color difference (< 2.7, ≤ 5.4 Δ*E*_ab_) was moderately unacceptable but still within a range considered for clinical use. The sum of all percentages across these three categories represents the quality range of shade matches that fall within industry tolerance for dentistry. Based on this, a new compound metric, termed *clinical pass rate*, was developed to evaluate the likelihood of a device achieving clinically acceptable results. The clinical pass rate was assessed against the 50/50% threshold, indicating the likelihood of a device achieving clinically acceptable shade selection.

Chi-square analysis was conducted to evaluate the significance of differences across devices for each of these categories. A 95% confidence level (*p* = 0.05) was used.

## Results

**Fig. 1:**
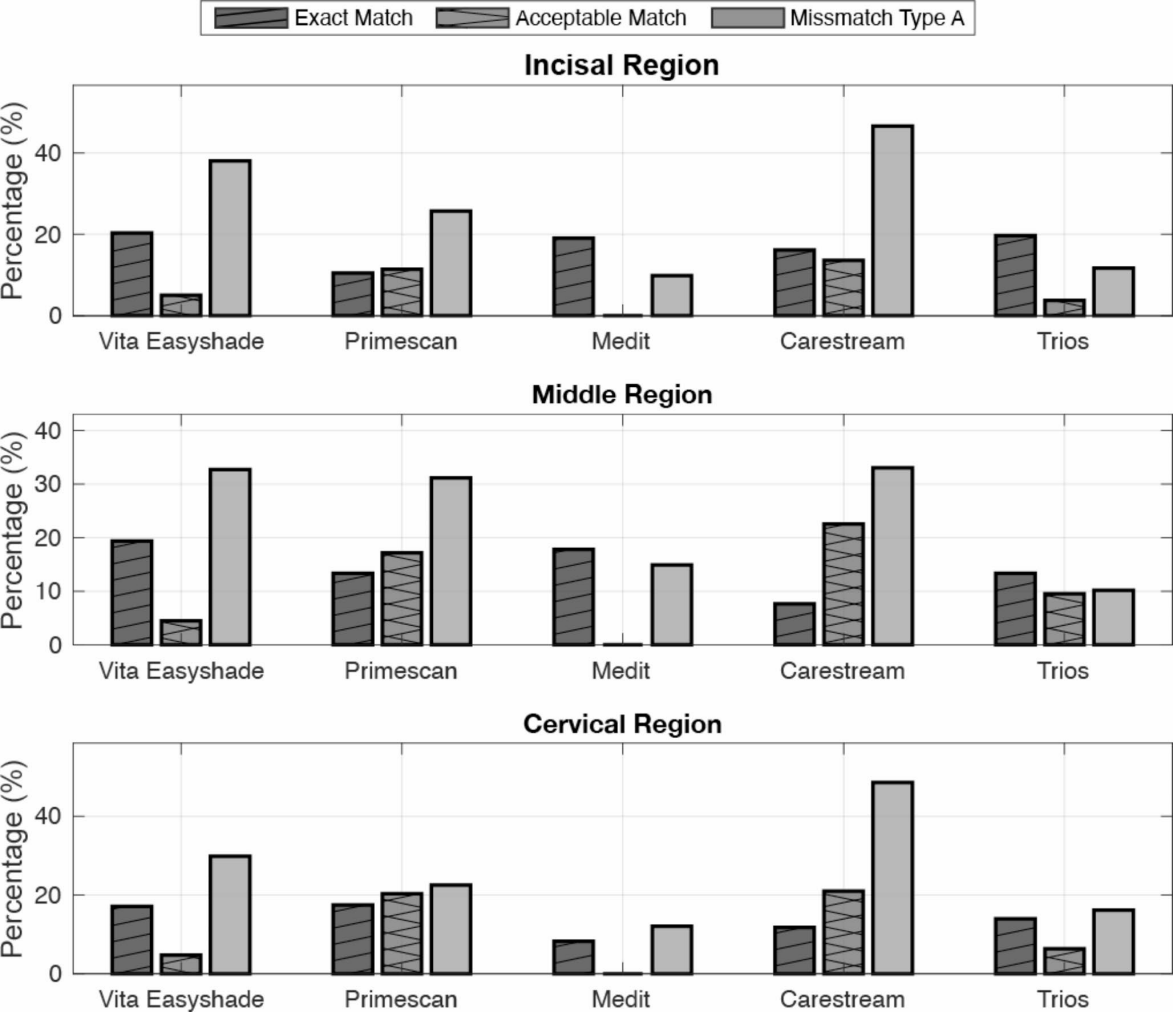
Percentages for all devices across incisal, middle, and cervical regions, displaying results of exact matches, acceptable matches (> 1.2, ≤ 2.7 ∆*E*_ab_), and Mismatch Type A (< 2.7, ≤ 5.4 ∆*E*_ab_). Results are shown for each device: Easyshade, Primescan, Medit, Carestream, and Trios

Results for all three regions for all included teeth and per each device, showing exact match, acceptable match and mismatch type A are shown in Fig. 1. In the incisal region, Easyshade achieved the highest Exact Match rate at 20.3%, followed by Medit at 19.0%, and Trios at 19.7%. Carestream recorded the highest rate for acceptable matches in this region at 13.71%, while Easyshade had 5.1%. For Mismatch Type A in the incisal region, Carestream led with 46.7%, followed by Easyshade at 38.1%. In the middle region, Easyshade also had the highest Exact Match rate at 19.4%, while Carestream had the highest percentage of acceptable matches at 22.5%. Carestream also exhibited the highest rate for Mismatch Type A at 33.0%. For the cervical region, Primescan achieved the highest Exact Match at 17.5%, while Carestream had the most acceptable matches at 21.0%. Carestream also led in Mismatch Type A in this region at 48.6%.

Averaged results across all three regions for all included teeth and per each device, showing Exact Match, Acceptable Match, Mismatch Type A, and overall clinical pass rate for each device are shown in Table 2. These results reflect the average across the three regions and three teeth per patient. Carestream achieved the highest clinical pass rate at 78.2%, followed by Easyshade with 63.5%, Primescan with 51.2%, Trios with 39.5%, and Medit with 31.3%. The Exact Match percentages ranged from 11.3% for Primescan to 22.1% for Trios. For the Acceptable Match rate, Carestream showed the highest percentage at 14.0%, while Medit had no acceptable matches. Mismatch type A was highest for Carestream at 47.7% and lowest for Medit at 10.7%. Differences in the clinical pass rate and across all categories for each device were statistically significant at the 95% confidence level (*p* = 0.05).

**Table 2:** Percentages of exact matches, acceptable matches (> 1.2, ≤ 2.7 ∆*E*_ab_), and mismatches type A (< 2.7, ≤ 5.4 ∆*E*_ab_) for each device, averaged across the cervical, middle and incisal regions. Clinical pass rate represents the sum of these categories, indicating likelihood of clinically agreeable shade selection by device. Chi-square test with 95% confidence level (*p* = 0.05) was used to evaluate statistical significance

Device	Exact match	Acceptable match	Mismatch Type A	Clinical Pass Rate
Carestream	16.6%	14.0%	47.7%	78.2%
Easyshade	20.3%	5.1%	38.1%	63.5%
Primescan	11.3%	12.3%	27.6%	51.2%
Trios	22.1%	4.3%	13.2%	39.5%
Medit	20.6%	0%	10.7%	31.3%
χ²	12.04	58.65	123.66	97.18
*p* = 0.05	*p* < 0.0171	*p* < 0.000	*p* < 0.000	*p* < 0.0001

## Discussion

This study aimed to evaluate the shade selection capabilities of various IOS, and one shade measurement device commonly mentioned in clinical research, from a practical perspective, rather than through the often-misinterpreted notion of *device accuracy*. The chosen approach focused on *percent correct shade identification*, grouping results into three clinically relevant categories: when a device’s selected shade either matched the observer’s selection, offered a clinically acceptable match, or was at least a moderately unacceptable match (Type A). To gauge clinical relevance, the sum of these categories presents a single measure referred to as the *clinical pass rate*.

Device performance differed significantly, as demonstrated by the chi-square test results across all categories. To interpret these findings, it is important to consider that historically in psychophysical studies designed to estimate visual thresholds, a 50% cutoff is often used as a standard [40]. Applying this concept to the present study, devices with a clinical pass rate at or above 50% should therefore be considered more reliable for shade selection, than those falling below this mark.

Carestream, with a clinical pass rate of 78.2%, clearly outperformed the other devices, positioning it as the most reliable option. It consistently provided clinically passable results, whether through exact matches or acceptable shade differences. Easyshade, which achieved a 63.5% pass rate, also performed well.

In contrast, both Primescan and Trios hovered near or below the 50% threshold, with Primescan just meeting the cutoff at 51.2%. This raises questions about the reliability of these devices when used without visual confirmation of shade selection. Medit, with a clinical pass rate of only 31.3%, demonstrated the lowest performance, indicating it may require alternative use strategies in clinical practice.

The visual ranking used in this study is grounded in established visual thresholds for clinical dentistry [41]. However, in practice, the acceptance or rejection of clinical restorations often depends on situational factors that cannot be entirely accounted for by visual thresholds. For instance, a clinical study by Ballard et al. [42] found that 94% of patients were either satisfied or extremely satisfied with an average shade match of 6.5 ∆*E*_ab_ thus exceeding the upper limit for clinical mismatch type A notably. Taking such findings into account, the inclusion of the Mismatch Type A category as part of the clinical pass rate can be justified with confidence.

As mentioned, there is growing interest in the shade selection capabilities of IOS devices [14, 17–19, 43]. In dental research, colorimetric accuracy has been defined as an instrument’s ability to provide color measurements identical to those of a reference device [44], though there is no consensus on what that reference should be. Some authors have proposed a radio-spectrometer for this purpose [43], while most dental researchers have designated the Vita Easyshade as their reference standard, likely due to its widespread availability and user-friendly operation. However, a recent multi-center study demonstrated that device performance was overshadowed by the choice of the color difference equation used [12]. Another study showed that color measurements from different devices labeled as spectrophotometers, when used on the same tooth samples, yielded incomparable CIELAB data [45]. For these reasons, the current study opted to use an expert observer as the reference instead of a color measurement device. Variations in methodologies across studies, coupled with ongoing confusion regarding the terms *accuracy* and *precision*, further complicate direct comparisons with the results of the present research.

This research employed a unique methodology aimed at providing insights that are practically relevant to the average dental practitioner, demonstrating that the shade selection abilities of certain IOS devices are comparable to, or even better than, those of a popular shade measurement device, and can therefore be reasonably trusted.

A key limitation of this study is the inclusion of only one expert observer, as a larger sample size of around 20 expert observers would have provided more robust and reliable results. Additionally, the intraoral scanners (IOSs) included in this study were not the latest generations of their respective models. The results might differ with newer generations, which could potentially offer improved performance.

Despite these limitations, the results of the present study demonstrate that IOS devices can indeed be reliable for shade selection, effectively meeting the demands of daily clinical practice.

## Conclusions

Within the limitations of this study, it can be concluded that IOS and traditional shade measurement devices show varying degrees of reliability for shade selection. The clinical pass rate, as used in this research, provides a practical metric for assessing device performance. Carestream exhibited the highest clinical pass rate followed by Easyshade, suggesting that these devices can be reasonably trusted in clinical practice. Other devices performed at or below the 50% mark, indicating that their use for shade selection should be considered more carefully. Despite these findings, it remains advisable to visually check the shade wherever possible, as visual assessment provides an additional layer of reliability.

## Data Availability

No datasets were generated or analysed during the current study.
